# Editorial: Innovations in phage biocontrol: advancing technology and applications

**DOI:** 10.3389/fmicb.2026.1837149

**Published:** 2026-04-14

**Authors:** Karen Fong, Hany Anany, Lin Chen, Thomas Brenner

**Affiliations:** 1 Agriculture and Agri-Food Canada, Summerland Research and Development Center, Summerland, BC, Canada; 2 Agriculture and Agri-Food Canada, Guelph Research and Development Center, Guelph, ON, Canada; 3 School of Chemistry, Chemical Engineering and Biotechnology, Nanyang Technological University, Singapore, Singapore; 4 Independent Researcher, Waterloo, ON, Canada

**Keywords:** biocontrol, CRISPR, pathogen, phage, phage application, phage biocontrol

## Introduction

Bacteriophages, viral predators of bacteria, have been well-studied for their capacity to control diverse pathogens and as promising alternatives to antibiotics across healthcare, food and agriculture sectors (Guo et al., 2020; Mahony et al., 2020; Pye et al., 2024). Phage selection for biocontrol applications typically includes isolation and characterization of novel phages that perform well during *in vitro* studies against target pathogen. Phage characterization involves bioinformatic analysis of the phage genome to confirm the absence of genes that could pose a risk during biocontrol applications (i.e., encoding for antimicrobial resistance, virulence, lysogeny). There is inherent value in such approaches as the discovery of novel phages further enrich and expand the pre-existing repertoire of known and characterized phages, particularly those possessing ultra-narrow host ranges whose biocontrol potential can be quite limited. Recently, there has been a surge of innovative approaches that capitalize on the existing diversity and ease of phage isolation and characterization. This Research Topic aims to showcase recent discoveries, developments and innovations in the utility of phage applications in diverse settings. Our Topic comprises nine articles, including six original research articles and three review articles. Topics span from perspectives on phage-based biocontrol in One Health, agriculture and healthcare to understanding phage components and systems to achieve optimal outcomes. The contributed papers present phage innovation in diverse applications across three themes: (1) discovery and characterization of novel phages; (2) phages and their components as alternative therapeutics; and (3) engineering and precision targeting using CRISPR-Phage systems.

## Discovery and characterization of novel phages

There were three manuscripts that focused on the isolation and characterization of phages for biocontrol applications. Hoang et al. provided comparative genomics and host interaction insights into two novel lytic phages, Macy and Sally, against carbapenem-resistant *Raoultella planticola*, an emerging nosocomial pathogen. Features attractive for biocontrol were described, including high burst sizes, broad host ranges and the ability to degrade biofilms. Additionally, Zhang et al. described the characterization of phages PSV6 and PSV3 against *Pseudomonas syringae* pv. *syringae* (Pss). They noted effective *in vivo* efficacy against Pss, potentiating their utility in biocontrol applications. Interestingly, Cheng et al. isolated a novel phage capable of cross-genus infection, vB_SmaS_QH3, that was able to lyse both *Stenotrophomonas maltophilia* and *Pseudomonas aeruginosa*. This phage exhibited significant antimicrobial activity against both hosts, along with good stability and infection kinetics.

## Phages and their components as alternative therapeutics

A large-scale bibliometric study on research into *Acinetobacter baumanii* phages revealed research hotspots and trends in this area, highlighting the trend toward targeting antimicrobial resistant (AMR) strains (Jiang et al.). Two reviews highlighted the growing need for alternative therapeutics to AMR. Wang et al. provided perspectives on the utility of phage therapy for intestinal bacterial infections. They elucidated clear benefits of using phages in such applications, including the promotion of microbiome stability, potential synergistic effects and the ability to treat AMR or multi-drug-resistant intestinal infections. Plat et al. further discussed application of phage in clinical and veterinary medicine and food in their narrative review. Additionally, they discussed the utility of phage-derived components such as endolysins and depolymerases for the replacement of antibiotics. To this end, Schwarzkopf et al. further elucidated the role of holins, phage-encoded hole-forming membrane proteins, in endolysin release. They provide evidence for the existence of a ring-shaped holin complex in phage T4 that enables endolysin release.

## Engineering and precision targeting using CRISPR-Phage systems

A novel predictive approach was developed by Ortiz-Cartagena et al. that utilizes the LAMP-CRISPR-Cas13a rapid-technique for detecting bacterial resistance mechanisms and predicting potential incompatibilities of therapeutic phages in the treatment of *Klebsiella pneumoniae*. Separately, Lee et al. cloned CRISPR-Cas12a genes into temperate phage λ and found it could selectively eliminate *E. coli* carrying target genomic sequences. Together, these two approaches advance the science on precision microbiome engineering tools to potentially overcome the inherent disadvantage of infection of non-target strains.

In conclusion, phage research, still in its infancy, is ever-evolving as there are still many avenues worth exploring. Fundamental work involving discovery and characterization of novel phages provides a solid foundation for application-driven work on studying phage-host interactions. As phages are being increasingly recognized globally for their therapeutic applications, further research is needed to enhance their potential across One Health, agriculture, food production and medicine.
